# A Case of Hydrophobia

**Published:** 1788

**Authors:** James Russel

**Affiliations:** Apothecary in London


### II. *A Case of Hydrophobia. By Mr.* James Russel, *Apothecary in London.*

ON the fifteenth of November, 1787, about eleven o'clock in the evening, I was desired to visit Francis Stanier, of Castle Street, Piccadilly. I found him in a state of great anxiety, and extremely restless, with a quick and small pulse, and complaining of considerable oppression about the præcordia.

The account the patient gave of himself was, that he was about sixty years of age; that he had had an ulcerated leg for the space of six years, which had healed towards the close of the year 1786; and that since that period he had been afflicted with rheumatism, and unable to follow his occupation, which was that of a smith; that for two or three days past he had felt violent pain in his left leg and thigh, which he had thought might be owing to his having cut some corns on his left foot; and that the day before I saw him he had found himself so ill, that he had been obliged to go to bed, and had not quitted it since.

Upon my asking him if he had felt any inclination to vomit, he started up suddenly, and, reaching for the pot before he answered me, said,

ſaid, "No, but that he then wanted to vomit," and, after ſeveral efforts, brought up a little mucus.

It appeared that he had taken no nouriſhment during the courſe of the day, and I was told that he had attempted to drink a little purl in the afternoon, but without being able to ſucceed. On receiving this information, I deſired that ſome table beer might be offered to him. To this he ſeemed to be extremely averſe; but, yielding to entreaty, he took a cupful of beer in his hand, and, after making ſeveral attempts to bring it to his mouth, at length threw it from him in a ſtate of the greateſt agitation.

This averſion to liquids, and the marks of horror he had diſplayed on being preſſed to drink, ſtruck me ſo forcibly as ſymptoms of hydrophobia, that, although I could not find, from my inquiries, there was any reaſon to believe he had been bit by any animal, I called upon Dr. Simmons, and mentioned to him my ideas of the nature of the caſe.

The patient that night was directed to take a bolus of muſk, thebaic extract, and cinnabar of antimony. This he ſwallowed, though not without extreme difficulty. A clyſter was alſo adminiftered; and while this was doing he was obferved to be exceffively agitated.

The next morning (Nov. 16) I was informed he had had a pretty good night; that his ftomach was compofed, and that he had had an evacuation by ftool; but as yet had not been able to drink any thing.

At breakfaft time the mafter of the houfe in which he lodged brought him a hot roll buttered, and a bafon of tea. He ate almoft the whole of the roll rather greedily, but pufhed back the tea, crying out at the fame time that it would be death to him to drink.

Dr. Jackfon, who faw him this day, about noon, found him walking about his chamber, and obferved that he anfwered fharply, and with great marks of agitation, when it was propofed to him to try to drink; but, upon being foothed and reafoned with, the patient was eafily perfuaded to try to take any thing that might be thought likely to relieve him, and did accordingly fwallow feveral fpoonfuls of a mixture prefcribed by Dr. Jackfon, confifting of camphorated julep, Hoffman's anodyne liquor, and the cordial confection. His pulfe at this period beat about an hundred ftrokes in a minute; his his ſkin felt rather cold. The ſtate of his tongue was moiſt and natural.

About eight o'clock in the evening Dr. Jackſon was met by Dr. Simmons, to whom I had written a note, informing him that the caſe appeared now to be a confirmed hydrophobia. The patient, at this period, complained much of ſpaſm at the upper part of his throat, which attacked him whenever he attempted to lay his head low, and obliged him frequently to raiſe it. His pulſe was ſtill of the ſame degree of quickneſs as at noon, and it was regular and of its natural fulneſs. His tongue was ſtill pretty clean, except at its baſis, where it was ſlightly furred. The ſtate of the fauces was examined; but in them no particular appearance could be diſcovered.

Timidity was ſtrongly marked in his countenance: he ſeemed to ſhrink within himſelf; frequently graſped the bed clothes; and when any mention was made of liquids, became ſuddenly agitated, and, with a voice expreſſive of diſtreſs and anger, begged us not to aſk him to drink. At this time he complained of a ſenſation in his throat, which he compared to ſtrangulation, and which occaſioned him to preſs

 the the external ſides of his fauces with his thumb and fingers.

Notwithſtanding the diſtreſs he laboured under, he was prevailed on to try to get down another ſpoonful of his mixture; but he obſerved to us at the ſame time, that he knew it would be impoſſible for him to ſwallow it unleſs he got out of bed. Accordingly he got up, and a table ſpoonful of the medicine being poured out, he took the ſpoon in his hand, trembling exceſſively, and putting it ſuddenly to his mouth, threw his head back, and, apparently with the utmoſt difficulty, ſwallowed ſome of the medicine. His general agitation, the wildneſs of his countenance, and tremor, were, for a few ſeconds, much increaſed.

He now ſate down on the edge of the bed, much agitated, and when he was a little more compoſed, it was propoſed to him that he ſhould put one of his hands into a baſon of water. His agitation evidently increaſed at the mention of this; but, upon being told that it might perhaps be of uſe to him, he conſented to try. A baſon filled with water was accordingly brought to him; but the moment his hand touched the water he ſnatched it back with marks of ſo much horror, that it was impoſſible ble juſt then to preſs him to the repetition of an experiment productive of ſuch evident diſtreſs.

About eleven o'clock the ſame evening the two phyſicians again viſited him, accompanied by Mr. Hunter and Mr. Everard Home. The patient was then in bed, apparently ſettled for the night. He ſeemed not to like to be diſturbed, and appeared, as he had done before, to be extremely timid and agitated; but, upon being a little more accuſtomed to his viſitors, and ſpoken to in a ſoothing manner, he became more compoſed, except upon particular topics, and theſe only ſuch as had a relation to fluids. He complained that ſpeaking brought on the uneaſy ſenſation, he had before ſpoken of, in his fauces; and it was obſerved that he ſeemed moſt affected by ſpeaking when he had raiſed himſelf a little from the bed.

He now replied to a variety of queſtions very deliberately and ſenſibly; talking, at laſt, with much compoſure even of liquids, when not connected with the idea of drinking. The idea of ſolids did not diſturb him ſo much.

He ſaid he was hungry, and ſhould reliſh food if he could ſwallow it with eaſe. The drier the food was the better, he ſaid, he liked it;

 but but the eating even ſome dry bread that was offered to him ſeemed to require a conſiderable degree of reſolution, a ſort of affected bravery, to get it down; and he appeared to chew it longer than he would otherways have done, but at laſt ſwallowed it tolerably well, conſidering it was dry.

It was propoſed to him that he ſhould try to ſwallow ſome jelly, and to this he readily aſſented. Some currant jelly was accordingly procured, and of this he twice ſwallowed a little, but evidently with much more difficulty and repugnance than he had ſhewn in ſwallowing the bread; for we obſerved that he ſnatched up the ſpoon and carried it to his mouth in a haſty manner, as if he had been ſummoning up reſolution to do a thing that was painful and difficult. When he had taken of it twice in this manner, he put the remainder by, ſaying he would keep it till the next day.

He was aſked to deſcribe what he had felt upon putting his hand into cold water. He ſaid it had felt to him colder than common, and had thrown inſtantly a ſenſation of cold over his whole body, which ſeemed to fly to the upper part of his throat. He had no ſoreneſs, he obſerved, in his throat when he ſwallowed, but a horrid ſenſation he was unable to deſcribe, and any chance of bringing it on threw him into great agitation.

When aſked whether he liked water or brandy beſt, he ſaid brandy, becauſe it was more palatable; but he obſerved that any thing liquid produced the uneaſy ſenſation, before mentioned, in his throat the moment it touched his lips. He obſerved alſo that the approach of any liquid was more offenſive to him when warm than cold, for the very ſteam offended him, and would bring on the uneaſineſs in his throat before he wetted his lips. This accounted for the repugnance he had ſhewn to the baſon of tea in the morning.

He ſpat very often, and ſeemed averſe to ſwallowing his ſaliva, which was ſmall in quantity and viſcid.

I had before endeavoured to learn whether there was any probability of his having been bit by a rabid animal; and the patient himſelf was now queſtioned on this ſubject, but in ſuch a cautious way as ſeemed the leaſt likely to excite in him any ſuſpicion relative to the motives for the inquiry. He told us that in the early part of his life he had been two or three times bitten by dogs in the hand, but he was certain that that nothing had been the matter with any of the dogs, as he had known them all long afterwards, and that the laſt time he had been bitten was at leaſt thirty years ago.

When we quitted him about midnight, it was agreed that he ſhould take a bolus, compoſed of conf. Damocr. ʒij. and of opium gr. iſs., and continue the uſe of his mixture. When my ſervant carried theſe medicines to him, he found him making violent efforts to vomit, and preſſing, at the ſame time, with his hands, each ſide of his throat. He likewiſe complained much of wind in his ſtomach, and was greatly agitated. He ſoon, however, became more compoſed, and was prevailed on to take the bolus, but not the mixture. He obſerved that the bolus felt warm and comfortable to his ſtomach, and made him belch. Soon after the ſtraining to vomit he ate a piece of bread rather voraciouſly. In the courſe of the night he ſlept a little; and the next morning found himſelf better, and thought he could drink a little purl.

At half paſt eleven o'clock (Nov. 17) he was again viſited by myſelf and the other gentlemen who had ſeen him the night before. We found him dreſſed, but lying on the bed covered with a blana blanket. Soon after we had entered the room he got up, and fate on the fide of the bed, telling us at the fame time that his throat was better, and that he was now able to drink. He had afked for fome purl before we came to him, and about a quarter of a pint of it ftill remained in the pot. This he drank in our prefence, but it feemed not to go down without fome difficulty, and his countenance fhewed that he felt himfelf happy when he had fwallowed it. It was remarked, however, that he did not feem to be fo much agitated when we talked of drinking, and of liquids, as he had been the night before, and his faliva was thought to be lefs vifcid.

When we had converfed with him a little while by the bed fide, he got up, and walked towards the table, to let us fee that he could now put his hands into cold water. This he accordingly did, and then wiped them dry with a towel. The water, he faid, ftill felt very cold, but not fo difagreeably fo as it had done the night before. In doing all this, however, he did not feem to be perfectly at his eafe, and it was obferved that he was much weaker than at our laft vifit. His pulfe was fo fmall as to be with difficulty felt, and fo irregular as to vary from from 80 to 100 ſtrokes in a minute. His tongue was moiſt, but whiter than it had hitherto been, and his eyes had a gloſſy appearance, as if covered with mucus.

About an hour after we had left him, as he was ſitting by the fire, he deſired the perſon who was with him to give him ſome jelly; but before this could be handed to him he fell from the chair, and Dr. Simmons, who came into the room immediately after, ſaw him expire in the courſe of a few minutes.

The body was examined the next morning by Mr. Hunter, in the preſence of the other gentlemen who had attended him. It was found to be uncommonly rigid. The ſternum was removed, and the œſophagus carefully expoſed throughout the whole of its extent. The ſtate of the fauces, trachea, and ſtomach, was alſo accurately examined.

In the cavity of the ſtomach ſome bile was found, together with a ſmall quantity of ſome other fluid. The inner ſurface of this viſcus was covered with a tough mucus, and near the entrance of the œſophagus were to be ſeen a few dots of extravaſated blood.

In the œſophagus there was no morbid appearance, if we except a thick mucus, which was was here rather in clots than lining the inner furface of the œfophagus as it did that of the ftomach. At the lower part of the œfophagus this mucus was tinged with a greenifh bile.

The gall bladder was very full of bile, and the colon and inteftines in general were much diftended with air.

This cafe, it is prefumed, may be added to the fmall number of inftances of fpontaneous hydrophobia recorded by medical writers. Of thofe inftances there is one publifhed in the Memoirs of the Royal Medical Society at Paris, which fo nearly refembles the one I have been relating, that I am perfuaded the reader will excufe my mentioning it briefly in this place.

The cafe in queftion occurred to M. Bonafos, Phyfician at Perpignan *. The patient was a maid fervant, thirty years old, who was feized with fymptoms of fever, and on the fifth day of her illnefs with hydrophobia, although no bite had preceded this fymptom. She complained of her throat, and of a difficulty of fwallowing; but no appearance of inflammation could be difcovered in the fauces. The dread of liquids

* Memoires de la Société Royale de Medecine. Années 1777 & 1778. 4to. Paris, 1780. p. 457.

ſoon became ſo great, that, although naturally of a mild and quiet diſpoſition, ſhe became irritated, and had violent convulſive motions when preſſed to drink. She was able, however, to ſwallow boluſſes of camphor and opium, and ſubmitted to the uſe of clyſters; but theſe remedies were ineffectual. The agitation and ſpaſms increaſed in violence; and her pulſe, from being full and ſomewhat hard, became ſmall, unequal, and even intermittent. At length, on the ſeventh day of the illneſs, and the ſecond from the commencement of the hydrophobia, the patient roſe up ſuddenly in her bed, in a ſtate of violent convulſion, and the moment after fell back dead.

*Swallow Street, Piccadilly,*
July 17, 1788.

