# Errata

**Published:** 1788

**Authors:** 


					?z(,o and for ' fate' read 4 fat.

				

